# Outbreak of *Neisseria meningitidis* serogroup B in Aragón, Spain, January to February 2025

**DOI:** 10.2807/1560-7917.ES.2025.30.14.2500206

**Published:** 2025-04-10

**Authors:** Cristina Nicolau Cano, Alba Gallego-Royo, Esteban Estupiñan Valido, Alejandra Perez Perez, Teresa Gimenez Julvez, Berta Maria Pilar Vela Iglesia, Maria Carmen Montaño Remacha

**Affiliations:** 1 Preventive Medicine Service, Miguel Servet Hospital, Aragón Health Service, Zaragoza, Spain; 2 Department of Public Health, Aragón Government, Zaragoza, Spain; 3 Microbiology Laboratory, Miguel Servet Hospital, Aragón Health Service, Zaragoza, Spain

**Keywords:** Meningitis, Meningococcal, Epidemics, Disease Outbreaks, Meningococcal Meningitis, Serogroup B

## Abstract

In January–February 2025, a community outbreak of *Neisseria meningitidis* serogroup B was reported in Aragón, Spain, with nine confirmed cases. This represents a 27-fold increase vs the previous 3 years. Five cases had respiratory co-infections; seven were attributed to complex CC213. Epidemiological investigations revealed a potential link to a school setting, though direct transmission could not be confirmed. These findings highlight the need to reinforce surveillance during periods of high respiratory virus circulation and in outbreaks involving non-4CMenB-covered variants.

Meningococcal disease (MD) is an acute infectious disease caused by *Neisseria meningitidis*, characterised by high morbidity and mortality and rapid clinical progression. Between January and February 2025, a community outbreak with nine cases of *N. meningitidis* serogroup B was detected in Aragón, an autonomous community located in north-eastern Spain with a population of ca 1.3 million inhabitants [1]. This event represents a notable increase in the expected number of serogroup B MD cases in the region, with an incidence in Aragón 27 times higher than that observed in the previous 3 years [2]. Here we describe the characteristics of the cases, potential epidemiological links and the prevention and control measures implemented.

## Case definitions and laboratory diagnostics

An upward trajectory of serogroup B MD has been observed in between 2022 and 2024 across several autonomous communities in Spain. In this outbreak, cases of serogroup B MD were reported to the Aragón epidemiological surveillance system. Cases were classified as possible, probable or confirmed based on the case definition from the MD Protocol of the Spanish National Epidemiological Surveillance Network (RENAVE) [3].

In addition to the marked increase in incidence, five cases were reported with concurrent respiratory viral infections, raising concerns about the potential role of viral co-infections in the epidemiological dynamics of the outbreak. In recent years, the implementation and increased use of nasopharyngeal swab testing for respiratory pathogens has notably improved the detection of viral co-infections, particularly in patients presenting with febrile syndromes.

The diagnosis of meningitis for each patient was established using PCR techniques (QIAstat-Dx Meningitis/Encephalitis panel). The suspicion of meningococcal sepsis was confirmed by a positive result with the BioFire FilmArray System Blood Culture Identification 2 (BCID2) panel on BD BACTEC blood culture vials after at least 4 h of incubation. This was followed by growth in culture media and antimicrobial susceptibility testing of the isolates.

All samples were initially processed at the reference hospitals and subsequently sent to the Spanish National Microbiology Center (CNM) for extended characterisation, including serogrouping, analysis of regional variants (RV) based on multilocus sequence typing (MLST) and the identification of specific porin proteins, clonal complex (CC) determination, antimicrobial susceptibility testing [4], molecular characterisation of key antigens (factor H binding protein (fHbp) and neisserial heparin binding antigen (NHBA)), and assessment of predicted vaccine coverage.

## Outbreak description

Nine cases of serogroup B MD were reported in early 2025, and all cases except one lived in the same city within the autonomous community of Aragón. In epidemiological week (EW) 1, two cases were reported. Subsequently, in EW 4, two cases of serogroup B MD were reported followed by two more in EW 5. In EW 6, two new cases appeared. The last notification appeared in EW 9. Seven were confirmed cases and two were probable, as *N. meningitidis* was identified in non-sterile site samples. Of the nine patients, four were males and five were females; five were aged 5 years and under, and four were aged 20 years and older. 

Notably, three cases (Cases 4, 5 and 6) initially presented with influenza-like symptoms, and received a diagnosis of influenza B through nasopharyngeal swab testing. One case diagnosed in EW6 (Case 7), following the epidemiological survey in the RENAVE protocol [3], presented a symptom onset date within 3 days of cases reported in EW 4 and 5, showed similar clinical presentation, and received a concurrent diagnosis of influenza A. Among the last two reported cases (Cases 8 and 9), the clinical presentation was characterised by shorter latency in symptom manifestation compared with previous cases. Case 8 also presented a concurrent respiratory infection with rhinovirus.

Of the nine confirmed cases, three had no documented history of vaccination against any meningococcal serogroup; all three were born before the year 2000. The remaining six cases (age range: 0–25) had received at least one dose of a meningococcal vaccine, including MenC, MenACWY or recombinant MenB, according to their age and regional immunisation schedule.

All seven confirmed cases were hospitalised, and their clinical context strongly supported the diagnosis of invasive MD. Case 3 presented with meningococcal sepsis, Cases 1, 2 and 7 with meningococcal meningitis and Cases 4, 8 and 9 with both conditions. In clinical terms, the first seven patients shared a similar progression, beginning with influenza-like symptoms followed by persistent fever and vomiting, neurological signs and petechial rash. All patients were discharged from hospital. The description of patients and microbiological data are shown in the Table.

**Table:** Characteristics of cases of serogroup B meningococcal disease, Aragón, Spain, January–February 2025 (n = 9)

Case	Age group (years)	Vaccine status	Days^a^	MenB serogroup results	Case classification^b^	Respiratory virus	Specimen type	Antimicrobial susceptibility testing^c^	Antigen characterisation	Vaccine coverage^d^
1	20–45	1 dose MenC + 1 dose Men ACWY	0	Serogroup B RV1:22 RV2:14 CC: 213	Confirmed	No	CSF	NA	fHbp: allele 65 / peptide 45 / variant 3 / subfamily A	Variant not covered by 4CMenB, but covered by MenB-fHbp
									NHBA: allele 33 / peptide 18	Variant not covered by 4CMenB
2	> 45	No	2	Serogroup B RV1:22 RV2:14 CC: 213	Confirmed	Not reported	CSF	NA	NA	NA
3	> 1–5	2 doses MenC	19	Serogroup B RV1:22 RV2:14 CC: 213	Confirmed	No	Blood	Rifampicin: 0.016 Ciprofloxacin: 0.002 Penicillin: 0.094 Ampicillin: 0.19 Cefotaxime: 0.004 Ceftriaxone: < 0.016	fHbp: allele 435 / peptide 366 / variant 3 / subfamily A	Variant not covered by 4CMenB; insufficient data to predict MenB-fHbp coverage
									NHBA: allele 33 / peptide 18	Variant not covered by 4CMenB
4	20–45	No	18	Serogroup B RV1:22 RV2:14 CC: 213	Confirmed	Influenza B	Blood	Rifampicin: 0.016 Ciprofloxacin: 0.002 Penicillin: 0.094 Ampicillin: 0.19 Cefotaxime: 0.004 Ceftriaxone: < 0.016	fHbp: allele 435 / peptide 366 / variant 3 / subfamily A	Variant not covered by 4CMenB; insufficient data to predict MenB-fHbp coverage
									NHBA: allele 33 / peptide 18	Variant not covered by 4CMenB
5	> 1–5	3 doses MenB + 2 doses MenC	17	Serogroup B RV1:22 RV2:14 CC: 213	Probable	Influenza B	Nasopharyngeal swab	NA	fHbp: allele 435/peptide 366 /variant 3/subfamily A	Variant not covered by 4CMenB; insufficient data to predict MenB-fHbp coverage
									NHBA: allele 33 / peptide 18	variant not covered by 4CMenB
6	> 1–5	3 doses MenB + 2 doses MenC	17	Serogroup B RV1:22 RV2:14 CC: 213	Probable	Influenza B	Nasopharyngeal swab	NA	fHbp: allele 435 / peptide 366 / variant 3 / subfamily A	Variant not covered by 4CMenB; insufficient data to predict MenB-fHbp coverage
									NHBA: allele 33 / peptide 18	Variant not covered by 4CMenB
7	0–1	2 doses MenB + 1 dose MenC	19	Serogroup B RV1:22 RV2:14 CC: 213	Confirmed	Influenza A	CSF	NA	fHbp: allele 65 / peptide 45 / variant 3 / subfamily A	Variant not covered by 4CMenB, but covered by MenB-fHbp
									NHBA: allele 33 / peptide 18	Variant not covered by 4CMenB
8	0–1	2 doses MenB + 1 dose MenACWY	37	Serogroup B RV1:74 RV2:1 CC: No assigned clonal complex	Confirmed	Rhinovirus	Blood	Rifampicin: 0.012 Ciprofloxacin: 0.003 Penicillin: 0.19 Ampicillin: 0.19 Cefotaxime: 0.004 Ceftriaxone: < 0.016	fHbp: allele 25 / peptide 25 / variant 2 / subfamily A	Variant not covered by 4CMenB, but covered by MenB-fHbp through cross-reactivity
									NHBA: allele 2086 / peptide 1937	Insufficient data to predict 4CMenB coverage
9	> 45	No	52	Serogroup B RV1:22 RV2:14 CC: pending	Confirmed	No	Blood	Rifampicin: 0.008 Ciprofloxacin: < 0.002 Penicillin: 0.094 Ampicillin: 0.094 Cefotaxime: < 0.002 Ceftriaxone: < 0.016	NA	NA

CSF: cerebrospinal fluid; fHbp: factor H binding protein; MenB: recombinant serogroup B vaccine; MenC: serogroup C meningococcal conjugate vaccine; MenACWY: serogroups A, C, W and Y meningococcal conjugate vaccine; NA: not available.

^a^ Days: interval in days from the symptom onset of the first reported case to that of each subsequent case.

^b^ Case classification was based on the RENAVE protocol [3]. Possible case: a person who meets the clinical criteria of the disease and presents with a biochemical test compatible with the disease. Probable case: a person who meets the clinical criteria of the disease and the epidemiological criterion. Confirmed case: a person who meets the clinical criteria of the disease and fulfils at least one of the laboratory diagnostic criteria. Each patient was classified based on the site of the positive sample: Cases with CSF positivity were considered meningococcal meningitis, those with blood cultures as meningococcal sepsis, and those with positive nasopharyngeal swabs were classified as probable cases.

^c^ Antibiotic susceptibility (MIC, minimum inhibitory concentration) expressed in micrograms per millilitre (µg/mL) [4].

^d^ Molecular analysis of vaccine-included antigens against meningococcal serogroup B (4CMenB and MenB-fHbp).

## Epidemiological investigation and outbreak response

In the first 2 months of 2025, the Men B incidence was 0.65 per 100,000 inhabitants in Aragón, higher than the national rate for the same period, which stands at 0.11 per 100,000 inhabitants. The age distribution of the cases in Aragón shows that five of nine cases belonged to the paediatric age group (≤ 5 years), as shown in the Table.

The epidemiological investigation identified a common link among Cases 3, 4, 5 and 6, all associated with the classroom of a school centre.

Following the confirmation of increased incidence, the Public Health Service issued an epidemiological alert to the regional healthcare system and implemented an active surveillance protocol for healthcare professionals. This included reminders about the clinical presentation of meningitis, with particular emphasis on the atypical forms observed during the outbreak, i.e. often associated with respiratory infections, to support timely and appropriate diagnostic testing. Individual control measures were also applied to each case. All patients received post-exposure chemoprophylaxis during hospitalisation according to RENAVE protocol [3] (e.g. a single intramuscular dose of 125 mg of ceftriaxone in cases under 18 years old). After recovery, all were referred to their primary care physicians for post-exposure vaccination with MenB and MenACWY vaccines. In addition, post-exposure chemoprophylaxis was administered to all close contacts identified (n = 153 people). 

## Microbiological investigation

Microbiological diagnosis was primarily based on direct microscopic visualisation, culture and molecular techniques. Gram-negative diplococci were only visualised in Case 1 and 4 by Gram staining, and cerebrospinal fluid (CSF) cultures were negative in Cases 3, 8 and 9. *Neisseria meningitidis* was isolated exclusively from blood culture samples.

Microbiological analysis from the National Microbiology Center confirmed that eight cases presented the same RV (RV1:22 and RV2:14) and seven cases with the same CC (CC:213); the CC of Case 9 was pending at publication. Cases 3, 4, 5 and 6 displayed identical molecular antigen profiles for fHbp; Cases 1 and 7 were also identical to one other. These six displayed an identical profile for NHBA. These similarities were observed despite a time interval of nearly 20 days between the symptom onset of Case 1 and that of the subsequent cases (Table 1).

## Discussion

Since 1997, a progressive decrease in incidence of MD has been documented in Spain, falling below 0.5 cases per 100,000 inhabitants in 2014. However, from 2015, a slight increase was observed, with peaks in 2018–19. A marked decline during the COVID-19 pandemic (2020–21) can be attributed to the implemented infection control and prevention measures, which were gradually lifted over time. Since 2022, overall incidence has risen, in line with neighbouring countries [4,18]. The national annual incidence rate in 2022 and 2023 was 0.23 and 0.55 cases per 100,000 inhabitants, respectively [5]. Meningococcal disease incidence in Spain has exhibited a cyclical pattern of fluctuations over time, with no identified common cause [6]. Given the detection of nine cases in Aragón in the first 2 months of 2025 resulting in an incidence of 54 cases per year (0.65 cases per 100,000 inhabitants), the projected annual incidence would represent a substantial increase compared to previous years (2 cases per year), potentially exceeding a 27-fold rise. 

The epidemiological surveillance services investigated potential epidemiological links and the temporal relationship between cases, identifying a connection of four cases notified in EW 4 with a school classroom. However, the time interval between the onset of symptoms in the first two cases and those linked to the school exceeded the usual 10-day incubation period, suggesting the absence of direct transmission. These findings raise the possibility of undiagnosed cases or asymptomatic carriers as the source of transmission, a hypothesis further supported by the molecular similarity observed among Cases 1–7.

It is noteworthy that the recombinant MenB vaccine currently included in the Spanish immunisation programme [9,10], 4CMenB, has not been shown to reduce carriage of *N. meningitidis* [11]. Although the vaccine was available for private purchase outside the hospital setting since 2015, 4CMenB was incorporated into the infant vaccination schedule in 2023 since the highest incidence of MD is typically observed in children under 5 years of age. In this outbreak, six of nine affected individuals have developed a breakthrough infection, as they had received at least one dose of a meningococcal vaccine. The three unvaccinated cases were all over 25 years of age, outside the target group of the MenB programme at the time of its implementation. Rather than indicating that the vaccine is not effective, these findings raise the need to assess whether the widespread use of 4CMenB could be exerting selective pressure, favouring the emergence of non-vaccine-covered variants. This hypothesis is supported by molecular data from the National Microbiology Center, which identified clonal complex CC213 in most cases—a strain not predicted to be covered by 4CMenB [12,13]. Although the alternative MenB vaccine, MenB-fHbp, appears to provide antigenic coverage against CC213, its use is currently authorised only for individuals from 10 years of age [14] and therefore the vaccine was not indicated in the majority of cases in this outbreak.

During the winter season of 2024/25, EW 4 represented the peak of acute respiratory infection (ARI) activity, with an incidence of 1,180.2 cases per 100,000 inhabitants [15]. This coincides with the cluster of four meningococcal cases linked to the school setting, suggesting a possible temporal association between heightened respiratory virus circulation and the occurrence of the outbreak. Three of the cases linked to the school setting had a concurrent influenza virus infection, suggesting a possible epidemiological association between both infections. Although previous studies have reported an increased risk of invasive meningococcal disease during an influenza infection [16,17], in vitro research does not support a direct biological mechanism enhancing *N. meningitidis* adherence or penetration of the nasopharyngeal mucosa [18]. In contrast, Case 8 presented with a sudden onset of typical meningitis symptoms in the absence of prior respiratory signs, and was co-infected with rhinovirus. Moreover, this case involved a different clonal complex than the other eight, suggesting a separate transmission chain or source.

This report is limited to a 2-month period, during which enhanced surveillance and public health measures were implemented to control the outbreak. In addition, no clear epidemiological link was identified for all confirmed cases, which may suggest undetected transmission chains. Moreover, whole genome sequencing (WGS) was not performed, limiting the ability to assess the genetic relatedness of the strains through phylogenetic analysis.

## Conclusion

This outbreak highlights the occurrence of multiple breakthrough infections, which appear to be associated with acute respiratory infections. The predominance of clonal complex CC213, not covered by the current 4CMenB vaccine, raises concerns about potential immune escape. These findings underscore the need to monitor emerging variants and consider their implications for vaccination strategies and outbreak control.
